# The moderating role of eating behaviour traits in the association between exposure to hot food takeaway outlets and body fatness

**DOI:** 10.1038/s41366-023-01290-9

**Published:** 2023-03-14

**Authors:** Jody C. Hoenink, Thomas Burgoine, Soren Brage, Nita Forouhi, Simon J. Griffin, Pablo Monsivais, Nicholas J. Wareham, Amy Ahern, Jean Adams

**Affiliations:** 1 MRC Epidemiology Unit, University of Cambridge School of Clinical Medicine, Box 285 Institute of Metabolic Science, Cambridge Biomedical Campus, Cambridge, CB2 0QQ UK; 2 Primary Care Unit, School of Clinical Medicine, University of Cambridge, East Forvie Building, Cambridge Biomedical Campus, Cambridge, CB2 0SR UK; 3 Elson S Floyd College of Medicine, Washington State University, Spokane, WA USA

**Keywords:** Risk factors, Nutrition disorders

## Abstract

**Background:**

Previous studies demonstrated a relation between takeaway outlet exposure and health outcomes. Individual characteristics, such as eating behaviour traits, could make some people more susceptible to the influence of the food environment. Few studies have investigated this topic. We aimed to investigate the moderating role of eating behaviour traits (cognitive restraint, uncontrolled eating and emotional eating) in the association between neighbourhood exposure to hot food takeaway outlets (hereafter referred to as takeaway outlets), and takeaway food consumption and adiposity.

**Methods:**

We used cross-sectional data from a cohort in Cambridgeshire, UK (The Fenland study). Takeaway outlet exposure was derived using participants’ residential address and data from local authorities and divided into quarters. The Three Factor Eating questionnaire (TFEQ-R18) was used to measure eating behaviour traits. Primary outcomes were consumption of takeaway-like foods (derived from food frequency questionnaire), and body fat percentage (measured using dual-energy X-ray absorptiometry).

**Results:**

Mean age of participants (*n* = 4791) was 51.0 (SD = 7.2) and 53.9% were female. Higher exposure to takeaway outlets in the neighbourhood and higher eating behaviour trait scores were independently associated with greater takeaway consumption and body fat percentage. Uncontrolled eating did not moderate the associations between takeaway outlet exposure and takeaway consumption or body fat percentage. The association between takeaway outlet exposure and takeaway consumption was slightly stronger in those with higher cognitive restraint scores, and the association between takeaway outlet exposure and body fat percentage was slightly stronger in those with lower emotional eating scores.

**Conclusion:**

Eating behaviour traits and exposure to takeaway outlets were associated with greater takeaway consumption and body fat, but evidence that individuals with certain traits are more susceptible to takeaway outlets was weak. The findings indicate that interventions at both the individual and environmental levels are needed to comprehensively address unhealthy diets.

**Trial registry:**

ISRCTN72077169

## Introduction

The obesogenic environment has been defined as the “sum of the influences that the surroundings, opportunities or conditions of life have on promoting obesity in individuals and populations” [1]. It contributes to the prevalence of energy-dense food consumption, low levels of energy expenditure, and high levels of overweight and obesity in the United Kingdom (UK) and elsewhere [2]. One aspect of the obesogenic environment is easy access to hot food takeaway outlets (e.g. outlets selling hamburgers and fried chicken; hereafter referred to as takeaway outlets). The prevalence of takeaway outlets has increased significantly in recent years [3, 4]. Evidence from the UK suggests that between 1990 and 2008, takeaway outlets increased by 43% in the most deprived areas, 50% in middle deprived areas and 30% in the least deprived areas [4].

While the evidence base is generally inconclusive [5, 6], studies have found that physical exposure to takeaway outlets around where people live and work is associated with unhealthy dietary behaviour and greater body weight [7–9]. More specifically, in a previous analysis of 5442 adults living in Cambridgeshire, UK, we found that exposure to takeaway outlets was associated with greater consumption of takeaway food, higher body mass index (BMI) and greater odds of obesity [7].

Despite the presence of high numbers of takeaway outlets in their environment, some individuals are still able to eat well and maintain a healthy weight [10]. The inconsistent evidence surrounding the influence of the food environment on dietary behaviours and obesity may partly be due to a failure to consider how individual differences in psychological traits interact with food environments [11, 12]. For example, some individuals may be more susceptible to takeaway outlets as environmental “cues” to eat than others. A previous study found a positive association between exposure to takeaway outlets and fast-food consumption, but only among children with higher scores for external eating (a psychological trait characterised by a stronger response to food cues in the environment such as the sight or smell of food) [11].

Eating behaviour traits are characteristics of individuals that influence behaviour and do not tend to fluctuate on a day-to-day basis [10]. External eating is one example; other examples include those commonly measured using the revised Three-Factor Eating Questionnaire (TFEQ-R18) [13]. The TFEQ-R18 assesses one’s conscious and constant effort to restrict food intake to achieve a desirable weight (cognitive restraint), having a heightened appetite (uncontrolled eating) and eating in response to negative emotions (emotional eating) [13, 14]. Previous studies found that higher uncontrolled and emotional eating scores were associated with lower diet quality [15, 16] and higher body weight [17–20]. The evidence with regards to cognitive restraint is more equivocal [21]. Overall, these eating behaviour traits may result in individual differences in the likelihood of responding to food cues in the environment, yet this has rarely been studied.

In this study we investigated the moderating role of eating behaviour traits in the relationship between exposure to nearby takeaway outlets, individual takeaway consumption and adiposity.

## Methods

### Study sample

Between 2005 and 2015, adults born between 1950 and 1975 were recruited to the Fenland Study from the population-based registers of general practices in Cambridgeshire, the UK [22]. Participants were asked to complete a general sociodemographic and lifestyle questionnaire, and a semiquantitative food-frequency questionnaire (FFQ) to assess habitual food consumption. Participants also attended a clinical research facility where measurements of body composition were made by trained researchers following standard operating procedures. During phase 1 of this ongoing population-based study, 46,024 individuals were invited to partake in the study and 12,435 adults were recruited (response rate of 27%) [22]. The current study used a sub-sample of the Fenland Study cohort (*n* = 4791), in whom eating behaviour traits were assessed using the TFEQ-R18. The Fenland study was approved by the Health Research Authority National Research Ethics Service Committee East of England-Cambridge Central.

### Exposure

The exposure of interest was the number of takeaway outlets within the residential neighbourhood, defined as a 1 mile radius around participants’ home address [7]. A description of the methods used for defining food environment exposures at home, as well as the validity of using secondary data sources, has previously been described [7, 23–25]. Briefly, participants’ home addresses were mapped by postcode using a geographic information system (ArcGIS 10, ESRI, Redlands, CA, USA) [24]. Data on food outlet (takeaway outlet and supermarket) locations were sourced from 10 local councils covering the study area in December 2011, and again mapped by postcode. Takeaway outlets were classified as those that sell hot food primarily for consumption off the premises, ordered and paid for at the cash register, with no wait staff and no or limited options for dining in. Takeaway outlets included both chains as well as local independent takeaway outlets.

### Outcomes

Two primary outcomes were used; consumption of energy dense “takeaway foods” which can be commonly obtained from takeaway food outlets, and adiposity, which in the present analyses were expressed as body fat percentage from dual-energy X-ray absorptiometry (DEXA) measurement. We included both takeaway-like food consumption as well as body fat percentage as outcomes as takeaway consumption is a proximal outcome of takeaway outlet exposure, and takeaway consumption has been shown to be positively associated to body weight and adiposity [26, 27]. Body fat percentage from DEXA as a measure of adiposity was preferred over the widely and previously used measure BMI [7, 28] because 1) BMI is an indirect measure of body fat compared to more direct approaches such as that from DEXA measurement, and 2) BMI is a less accurate obesity classification method. A previous study found that BMI misclassified 25% men and 48% women compared to DEXA measurement of body fat percentage, leading to a significantly underestimated prevalence of obesity [29].

Takeaway consumption was measured using data from a food frequency questionnaire. We calculated intake (g/day) of pizza, burgers, fried fish, and French fries. Together, these foods provide a marker of takeaway-like food consumption (g/day) referred to here as “takeaway consumption”. Procedures for how body fat percentage was measured are described elsewhere [30]. Briefly, participants attended a clinical research facility after an overnight fast, where height (cm) and weight (kg) were measured by trained research assistants. Total body fat mass (g) was determined with standard imaging and positioning protocols.

As secondary outcomes, we included fat mass index (FMI) as a different expression of the DEXA data as well as BMI to allow for comparison with previous results from the same cohort [7]. FMI was calculated as fat mass (kg) divided by height squared (m^2^), and BMI was calculated as weight (kg) divided by height squared (m^2^).

### Moderators

As eating behaviour traits were introduced part way through the Fenland Study, this was only available in a sub-sample of the Fenland Study cohort (*n* = 4791). Eating behaviour traits were measured using the TFEQ-R18 [13]. This questionnaire assesses cognitive restraint, uncontrolled eating and emotional eating using 18 items each on a 4-point Likert scale. The structural validity of the TFEQ-18 in the current sample was assessed using confirmatory factor analysis. This confirmed that the 18 items loaded onto the three factors of cognitive restraint (Cronbach’s alpha 0.75; 6 items), uncontrolled eating (Cronbach’s alpha 0.85; 9 items), and emotional eating (Cronbach’s alpha 0.87; 3 items). As in a previous study [31], raw scores were transformed to a 0–100 scale [((raw score − lowest possible raw score)/possible raw score range) × 100]. Higher scores in their respective scales are indicative of greater cognitive restraint, uncontrolled eating, or emotional eating.

### Covariates

We included covariates that were hypothesized to be associated with both takeaway outlet exposure as well as the outcomes but not on the causal pathway. Covariates captured in the Fenland Study general lifestyle questionnaire included sex, age, age at completion of full time education, occupation social class (categorized as professional, intermediate or working class [32]) and total combined annual household income (<£20,000, £20,000–£39,999, ≥£40,000). We also included the number of supermarkets within home neighbourhoods defined within a 1-mile Euclidean buffer [7].

### Statistical analyses

We reported sample characteristics using the mean (SD) for normally distributed continuous variables, and median (IQR) for skewed continuous variables. We reported sample characteristics for categorical variables as *n* (%). As outliers can negatively impact regression analyses, we adjusted outliers to the mean ± 3 SD. Data on eating behaviour traits were collected in the latter years (2011–2015) of phase 1 of the Fenland Study (2005–2015).

**Fig. 1:**
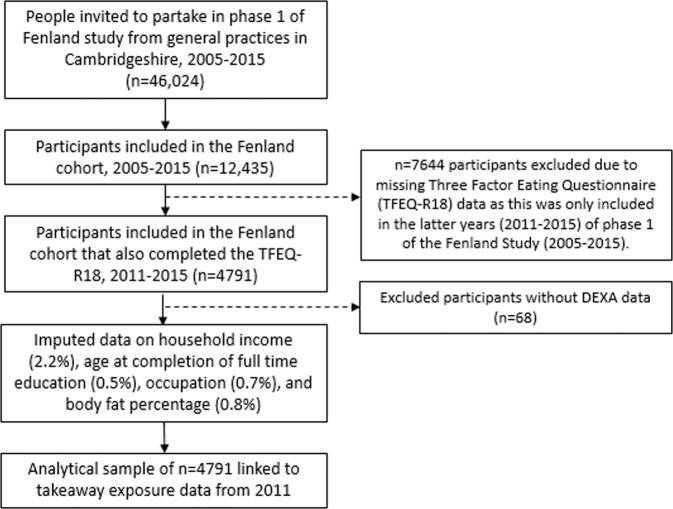
Flowchart of participant inclusion. Flowchart of participant inclusion to the Fenland phase 1 study up to the analytical sample included in the current study.

Figure 1 depicts the flow of participants from our full to analytic sample. We excluded participants without eating behaviour trait data. Subsequently, we excluded participants that were not measured using a DEXA machine (*n* = 68; due to one study site not having a DEXA machine for the first period of data collection) [30]. Missing data on other variables of interest ranged from 0% (e.g. age and sex) to 2.2% (i.e. household income), leading to a sample of *n* = 4507 (94%) with complete data. Given that we had missing data in at least 1 variable (including body fat percentage) for more than 5% of participants, and there was a reasonable likelihood that data were missing at random, we followed the recommendations of Jakobsen et al. [33], and used multivariate imputation by chained equations (MICE) to impute covariates with missing data as well as body fat percentage (seed set at 1234 and 20 imputed datasets). MICE is a multiple imputation technique that is flexible and can handle categorical as well as continuous variables [34].

We divided exposure to takeaway outlets into quartiles for analysis for two reasons. First, previous analyses showed that the association between takeaway outlet exposure and takeaway consumption is non-linear [7]. Second, environmental exposure misclassification may have occurred as the number of takeaway outlets and the outcomes (takeaway consumption and body fat percentage) were measured at different time points (2011 and 2005–2015, respectively). Operationalising takeaway outlet exposure in this way minimises potential misclassification as assignment to quarters is less sensitive to unmeasured food environment change over time [28]. We used multiple linear regression models to investigate associations between exposure to takeaway outlets and the outcomes takeaway consumption and body fat percentage, and between the three eating behaviour traits and outcomes. We adjusted all models for age, sex, household income, age at highest attained educational level, occupational social class and count of supermarkets in home neighbourhood.

We investigated effect modification by adding multiplicative interaction terms (eating behaviour traits x takeaway outlet exposure quartile) to these models. We tested for evidence of interaction using the *F-*test (for continuous outcomes) and pooling the *p*-values of the separate multiple imputed datasets using the Median P Rule [35]. In order to reduce multicollinearity between predictors and interaction terms, eating behaviour traits were mean centred [36]. In line with Aiken and West [37], we probed statistically significant interactions by estimating the conditional effect of takeaway outlet exposure at various values of eating behaviour traits (i.e. mean±1 SD). Considering the lower statistical power of interaction testing in non-experimental research, we set statistical significance at a two-sided α level of 0.10 [11]. We assessed all other results with a two-sided α level of 0.05 and all analyses were conducted in STATA v16.

## Results

Mean age of participants was 51.0 (SD 7.2) years and 53.9% were female (Table 1). In the overall analytic sample, median takeaway consumption was 30.0 g/day (IQR 17.5–47.5) and mean body fat percentage was 33.3 (SD 9.2). While unadjusted differences between quarters of takeaway outlet exposure were small, participants in the fourth (highest) quarter of takeaway outlet exposure consumed the least takeaway foods (27.6 g/day (IQR 10.5–41.2)), had the lowest mean body fat percentage (32.6% (95%CI 9.3) and the lowest BMI (26.2 kg/m^2^ (95%CI 4.4). Median exposure to takeaway outlets in the neighbourhood environment was 2.0 (IQR 0.0–12.0). The analytic sample (*n* = 4791) is similar to the overall Fenland Study sample (*n* = 12,325) (Supplementary Table 1).

**Table 1:** Characteristics of the unimputed Fenland analytical sample (*n* = 4791) by quarters of takeaway outlet exposure.

		Mean (SD), median; p25-p75, or *N* (%)					
		*N*	Q1 (lowest exposure; *n* = 1348)	Q2 (*n* = 1267)	Q3 (*n* = 1167)	Q4 (highest exposure; *n* = 1009)	Total sample (*n* = 4791)
Age, years (mean (SD))		4791	51.1 (6.9)	50.7 (7.2)	50.7 (7.3)	51.5 (7.4)	51.0 (7.2)
Female (*n* (%))		4791	757 (56.2%)	681 (54.8%)	613 (52.5%)	530 (52.5%)	2581 (53.9%)
Age at completion of full time education, years (mean (SD))		4767	18.9 (3.7)	18.7 (3.4)	18.1 (3.6)	20.2 (4.3)	19.0 (3.8)
Annual household income, £ (*n* (%))	<20,000		130 (9.9%)	150 (12.0%)	182 (16.1%)	629 (13.5%)	629 (13.5%)
	20,000–39,999	4685	404 (30.7%)	383 (30.6%)	433 (38.2%)	1512 (32.2%)	1512 (32.2%)
	≥40,000		783 (59.5%)	717 (57.4%)	518 (45.7%)	2544 (54.3%)	2544 (54.3%)
Occupational social class (*n* (%))	Working class		229 (17.0%)	250 (19.9%)	301 (26.0%)	980 (20.6%)	980 (20.6%)
	Intermediate	4759	311 (23.2%)	248 (19.7%)	251 (21.6%)	985 (20.7%)	985 (20.7%)
	Professional		800 (59.7)	761 (60.4%)	608 (52.4%)	2794 (58.7%)	2794 (58.7%)
Takeaway outlets in home neighbourhood, count (median (IQR))		4791	0.0 (0.0–0.0)	2.0 (1.0–2.0)	7.0 (5.0–9.0)	26.0 (21.0–37.0)	2.0 (0.0–12.0)
Supermarkets in home neighbourhood, count (median (IQR))		4791	0.0 (0.0–0.0)	0.0 (0.0–1.0)	2.0 (1.0–2.0)	6.0 (4.0–10.0)	1.0 (0.0–2.0)
Emotional eating, score ranging from 0–100 (mean SD))		4791	35.5 (26.8)	34.6 (28.2)	34.7 (28.6)	33.8 (26.7)	34.7 (27.6)
Uncontrolled eating, score ranging from 0–100 (mean (SD))		4791	30.1 (17.0)	30.1 (17.2)	30.2 (18.1)	29.1 (17.3)	29.9 (17.4)
Cognitive restraint, score ranging from 0–100 (mean (SD))		4791	42.2 (19.6)	40.7 (20.1)	40.5 (19.4)	41.6 (19.5)	41.3 (19.6)
Takeaway food consumption, g/day (median (IQR))		4791	30.0 (16.4–47.5)	30.4 (18.8–47.4)	31.1 (18.8–49.3)	27.6 (10.5–41.2)	30.0 (17.5–47.5)
Body fat percentage (mean (SD))		4754	33.2 (9.4)	33.0 (8.8)	34.4 (9.4)	32.6 (9.3)	33.3 (9.2)
Fat mass index (mean (SD))		4754	0.9 (0.4)	0.9 (0.3)	1.0 (0.4)	0.9 (0.3)	0.9 (0.4)
Body mass index (mean (SD))		4791	26.8 (4.8)	26.9 (4.9)	27.8 (5.1)	26.2 (4.4)	27.0 (4.8)

### Associations with takeaway consumption and body fat percentage

After adjustment for sociodemographic characteristics, compared to those living in neighbourhoods in the lowest quarter of takeaway outlet exposure, living in neighbourhoods in the highest two quarters was associated with greater takeaway consumption and body fat percentage (Table 2).

**Table 2:** Associations of exposure to takeaway outlets and eating behaviour traits with consumption of takeaway food and body fat percentage in the imputed Fenland Study sample (*n* = 4791).

	Takeaway consumption (g/day)		Body fat %	
	β	95% CI	β	95% CI
Takeaway outlet exposure quarter				
Q1 (*N* = 1348; 0 outlets)	Ref.	Ref.	Ref.	Ref.
Q2 (*N* = 1267; 1–2 outlets)	0.6	−1.4; 2.6	0.3	−0.3; 0.9
Q3 (*N* = 1167; 3–12 outlets)	**2.6**	**0.5; 4.8**	**1.8**	**1.2; 2.5**
Q4 (*N* = 1009; 13–51 outlets)	**4.0**	**0.3; 7.6**	**1.8**	**0.7; 2.9**
Cognitive restraint^a^	−**2.2**	−**2.6;** −**1.8**	**0.2**	**0.1; 0.3**
Emotional eating^a^	**0.6**	**0.4; 0.9**	**0.9**	**0.8; 1.0**
Uncontrolled eating^a^	**1.3**	**0.9; 1.7**	**1.1**	**0.9; 1.2**

Bold values are statistically significant at *p* < 0.05.

All models were adjusted for age, sex, household income, occupation, age at highest educational qualification and counts of supermarkets in home neighbourhoods.

*B* unstandardized beta regression coefficient, *95% CI* 95% Confidence Interval, *Ref.* Reference.

^a^Eating behaviour traits are presented on a scale from 0 to 100 in increments of 10 units.

Emotional eating and uncontrolled eating were positively associated with both takeaway consumption and body fat percentage (Table 2). For example, a 10-point higher uncontrolled eating score was associated with a 1.1 (95%CI 0.9; 1.2) higher body fat percentage. A higher cognitive restraint score was associated with lower takeaway consumption, but greater body fat percentage. The results for FMI and BMI are similar to those found for body fat percentage (Supplementary Table 2), as are the results using a complete-case analysis (Supplementary Table 3).

### Takeaway consumption and body fat percentage according to eating behaviour traits, socio-demographic characteristics and takeaway outlet exposure

**Fig. 2:**
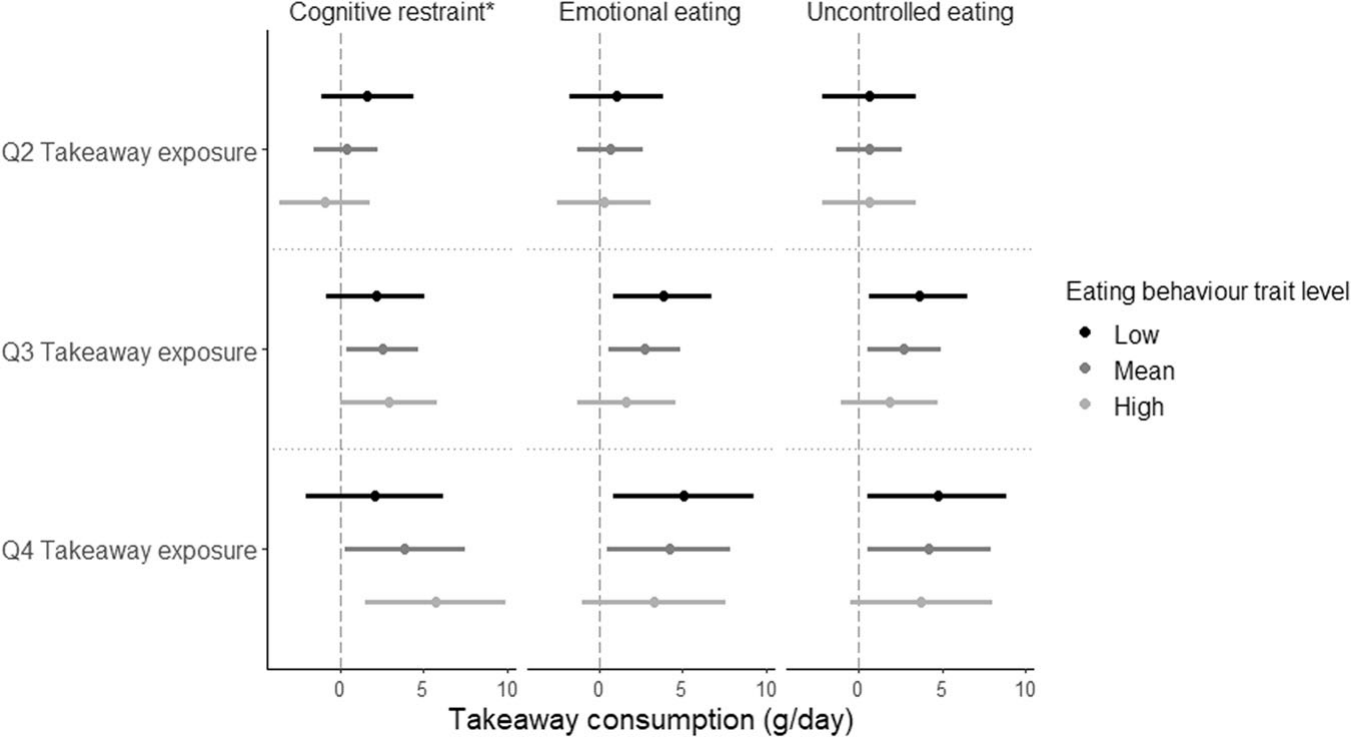
Beta regression coefficient of the association between takeaway outlet exposure and takeaway consumption stratified by eating behaviour traits. Beta regression coefficient and 95% coefficient interval in the association between takeaway outlet exposure (taking Q1 as the reference group) and takeaway consumption stratified by eating behaviour traits. Asterisk indicates a statistically significant interaction by eating behaviour trait in the association between takeaway outlet exposure and takeaway consumption.

**Fig. 3:**
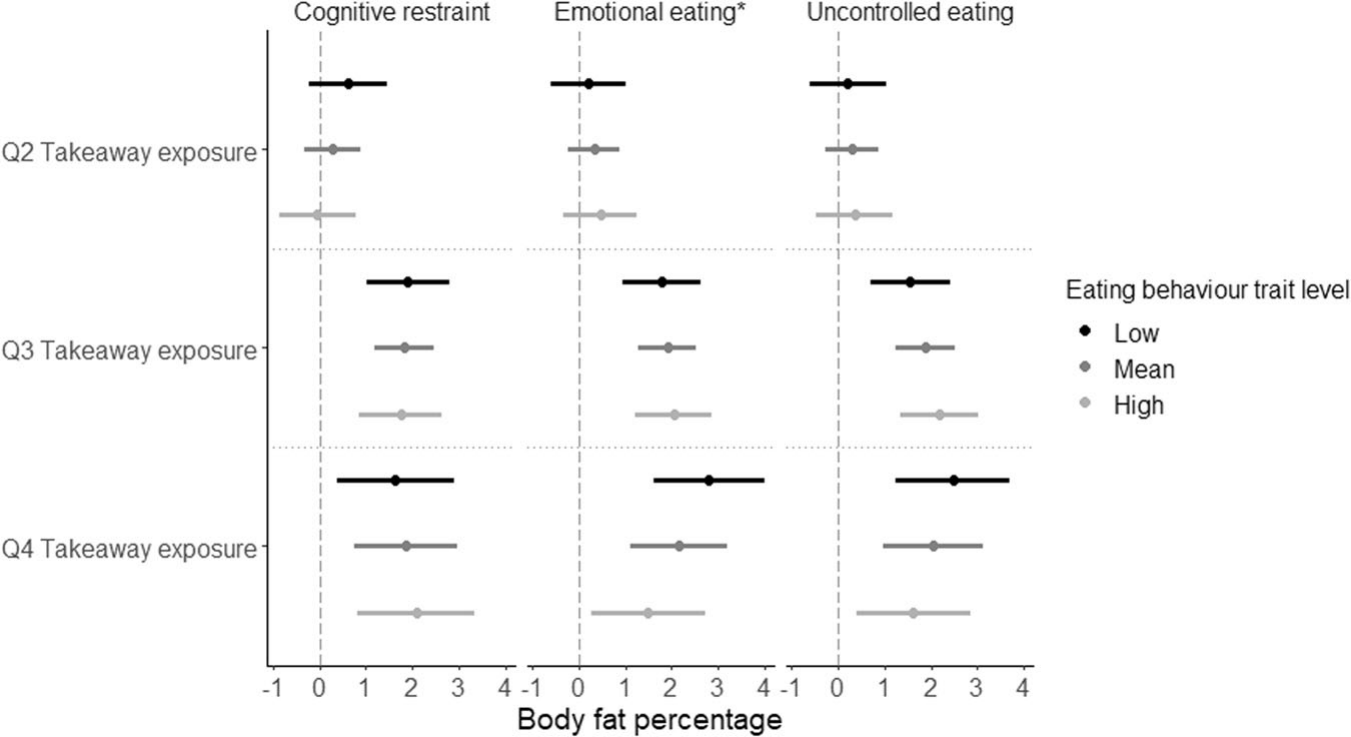
Beta regression coefficient of the association between takeaway outlet exposure and body fat percentage stratified by eating behaviour traits. Beta regression coefficient and 95% coefficient interval in the association between takeaway outlet exposure (taking Q1 as the reference group) and body fat percentage stratified by eating behaviour traits. Asterisk indicates a statistically significant interaction by eating behaviour trait in the association between takeaway outlet exposure and body fat percentage.

Figures 2 and 3 display the associations between takeaway outlet exposure, and takeaway consumption and body fat percentage stratified by eating behaviour traits. Only cognitive restraint moderated the association between takeaway outlet exposure and takeaway consumption (*p*-value F-statistic_cognitive restraint_ = 0.03, *p*-value F-statistic_emotional eating_ = 0.72 and *p*-value F-statistic_uncontrolled eating_ = 0.80 and). While for body fat percentage, both emotional eating and uncontrolled eating seem to have a similar effect on the association between takeaway outlet exposure and body fat percentage, only emotional eating statistically significantly moderated this association (*p*-value F-statistic_cognitive restraint_ = 0.40, *p*-value F-statistic_emotional eating_ = 0.05 and *p*-value F-statistic_uncontrolled eating_ = 0.14).

**Fig. 4:**
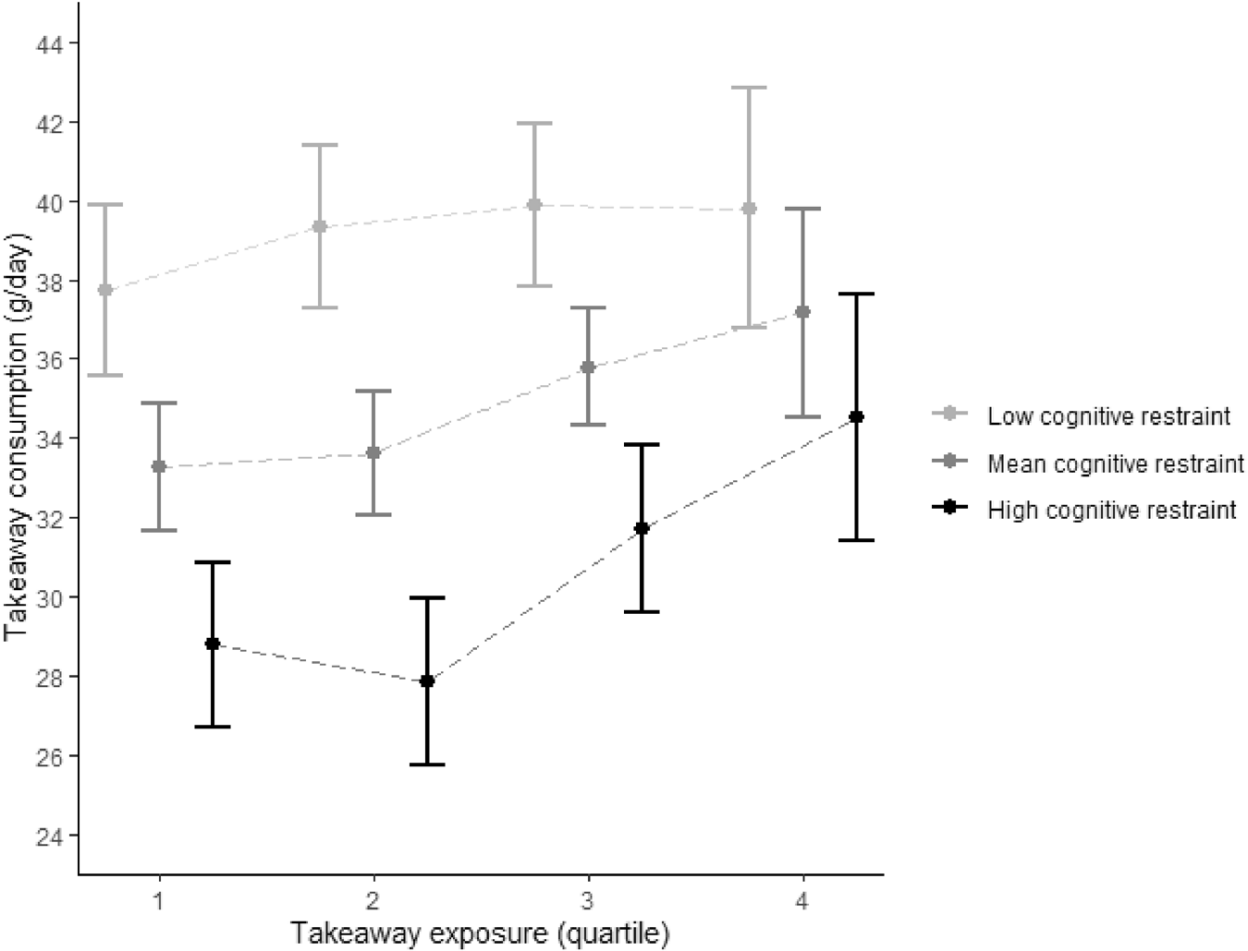
Mean takeaway consumption per quartile of takeaway outlet exposure stratified by cognitive restraint. Mean and 95% confidence interval takeaway consumption per quartile of takeaway outlet exposure in the Fenland Study (*n* = 4791) stratified by cognitive restraint (low = 1SD – mean; high = 1SD + mean) adjusted for age, sex, household income, occupation, age at highest educational qualification and counts of supermarkets in home neighbourhoods.

**Fig. 5:**
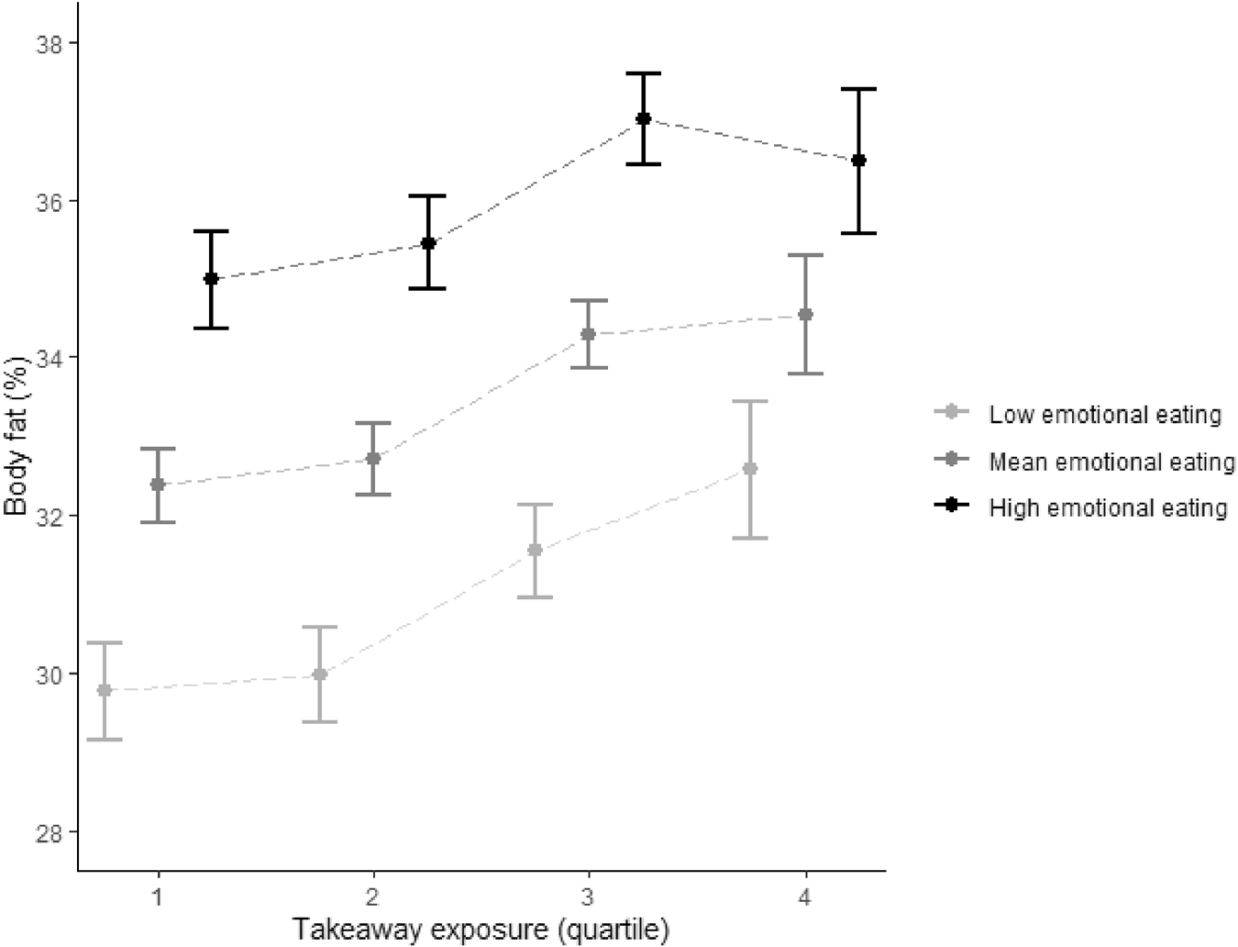
Mean body fat percentage per quartile of takeaway outlet exposure stratified by emotional eating. Mean and 95% confidence interval body fat percentage per quartile of takeaway outlet exposure in the Fenland Study (*n* = 4791) stratified by emotional eating (low = 1SD – mean, high = 1SD + mean) adjusted for age, sex, household income, occupation, age at highest educational qualification and counts of supermarkets in home neighbourhoods.

To gain a better understanding of the moderating role of eating behaviour traits, Figs. 4 and 5 show the mean takeaway consumption or body fat percentage per quartile of takeaway outlet exposure stratified only for the models with statistically significant interaction terms. At all levels of takeaway outlet exposure, those with lower cognitive restraint scores had greater takeaway food consumption (Fig. 4). The association between takeaway outlet exposure and takeaway consumption was not statistically significant for participants with low cognitive restraint scores, and strongest in participants with high cognitive restraint scores (Fig. 4 and Supplementary Table 4). Furthermore, while the difference in takeaway consumption between low, mean and high cognitive restraint was statistically significant in the first three quarters of takeaway outlet exposure (as indicated by the non-overlapping 95% confidence intervals), this was no longer the case in quarter 4 of takeaway outlet exposure. In other words, the association between cognitive restraint and takeaway consumption present at lower levels of takeaway exposure was absent at the highest level of takeaway outlet exposure.

The results in Fig. 5 indicate that at all levels of takeaway outlet exposure, those with higher emotional eating scores had a greater body fat percentage than those with lower emotional eating scores. We also found that the positive association between takeaway outlet exposure and body fat percentage was present at all levels of emotional eating, but strongest for low emotional eaters. For example, low emotional eaters most exposed to takeaway outlets had a 2.8% (95%CI 1.6; 4.0) greater body fat percentage than those least exposed to takeaway outlets (Supplementary Table 4). For high emotional eaters, this difference was 1.5% (95%CI 0.3; 2.7). Similar to cognitive restraint, the absolute difference in body fat percentage between those with low, mean and high emotional eating was smallest at highest levels of takeaway outlet exposure.

## Discussion

In a sample of almost 5000 UK adults, we found that takeaway outlet exposure as well as emotional eating and uncontrolled eating were positively associated to both takeaway consumption and body fat percentage. We also found that cognitive restraint was negatively associated with takeaway consumption, but positively associated with body fat percentage. Eating behaviour traits partly moderated the association between takeaway outlet exposure and takeaway consumption and body fat percentage. Specifically, uncontrolled eating did not moderate the association between takeaway outlet exposure, and takeaway consumption and body fat percentage. Cognitive restraint moderated the association between takeaway outlet exposure and takeaway consumption, and emotional eating moderated the association between takeaway outlet exposure and body fat percentage. Namely, the association between takeaway outlet exposure and takeaway consumption was somewhat stronger for individuals with high cognitive restraint scores compared to lower cognitive restraint scores. Furthermore, the association between takeaway outlet exposure and body fat percentage was somewhat stronger for individuals with low emotional eating scores compared to higher emotional eating scores. However, these effects were small in all cases.

While the evidence base with regards to the association between exposure to food outlets in residential neighbourhoods and dietary behaviours is generally mixed [5, 6], the current study findings are in line with previous studies conducted in the UK [8, 9, 38]. As here, previous studies have found that having higher emotional and uncontrolled eating tendencies were associated with lower diet quality [15, 16] and higher body weight [17–20]. Some previous studies also found that higher cognitive restraint scores were associated with higher diet quality [39] and lower energy intake [18]. Comparing our interaction findings to the wider literature is difficult as very few studies have investigated the influence of psychological factors in the association between the food environment, and dietary measures or health outcomes in adults. One previous study found that the positive association between takeaway outlet exposure and takeaway consumption was stronger amongst those with higher reward sensitivity (i.e. being prone to experiencing a positive effect in response to incentives or appetitive stimuli) [12].

We found some evidence of effect modification by cognitive restraint and emotional eating, but not uncontrolled eating. Furthermore, the moderating role of emotional eating and cognitive restraint on the association between takeaway outlet exposure, and takeaway consumption and adiposity was relatively small. As the evidence for interaction was not consistent across potential moderators and outcomes, and given the number of tests performed, it is possible that our findings are based on chance. The small moderating role of cognitive restraint and emotional eating is, however, not surprising as takeaway outlets are responsible for only a small amount of the variance in adiposity. Also, the direction of the effect modification is not surprising, which we explain in the following paragraphs.

Cognitive restraint was negatively associated with reported takeaway consumption, but positively associated with body fat percentage. Given the positive association between takeaway consumption and body weight [26, 27], we expected the associations between cognitive restraint, and takeaway consumption and body fat to be in the same direction (unless differential bias by restraint was present in the reporting of food intake). The current study findings together with previous findings suggest that the association between cognitive restraint and adiposity is likely bi-directional. Previous longitudinal studies found that high baseline adiposity or BMI was more likely to be associated with increased, rather than decreased, cognitive restraint [40, 41], suggesting that exerting cognitive restraint is likely to be a consequence of increased body weight instead of a cause. This bi-directionality may explain our study findings; we found that the association between takeaway outlet exposure and takeaway consumption (but not body fat percentage) was somewhat stronger among individuals with higher cognitive restraint scores. Individuals with high cognitive restraint scores may be actively trying to eat less, which is more difficult in areas with high takeaway outlet exposure compared to lower exposure.

In the current analysis, emotional eating was positively associated with both takeaway consumption and body fat percentage. Contrary to the aforementioned study [12], the results seem to suggest that the association between takeaway outlet exposure and body fat percentage is stronger for individuals with *lower* emotional eating scores, albeit at lower absolute levels of adiposity. This finding may suggest that the close environment matters more for those with *less* individual tendencies of partaking in unhealthy dietary behaviours. The findings may additionally suggest that high emotional eaters will find takeaway food if they want it, regardless of the local density of takeaway outlets. Previous studies found that eating behaviour traits are partly hereditary [42, 43], and that genetic risk for obesity was positively associated with emotional eating [31]. In a previous analysis conducted in the Fenland cohort, we found that the association between takeaway outlet exposure and BMI for those with a high and low genetic risk of obesity did not differ statistically significantly [28]. Nevertheless, we did find a somewhat weaker association between the food environment and BMI among those with higher genetic risk of obesity compared to those with lower genetic risk, indicating that the local environment mattered *less* for those at high individual risk of obesity. We argued in that study that the findings are consistent with Rothman’s component cause model [44], which suggests that for multiple cause outcomes, the strength of one causal factor is influenced by the relative prevalence of other causal factors [28].

The current study results suggest that those exposed to the most takeaway outlets are at an increased risk of adiposity than those exposed to the least takeaway outlets. Thus, intervening on eating behaviour traits alone to improve health outcomes will not remove the negative influence the current obesogenic environment has on these health outcomes. The reverse is also true; intervening only on the food environment will not remove all individual differences in dietary behaviours and adiposity. As such, there is a need to implement integrated strategies addressing both individual and environmental influences on dietary behaviours. Multiple interventions at different levels of influence need to be implemented to reduce unhealthy dietary behaviours and obesity levels, including downstream approaches targeting individuals most at risk (e.g. mindfulness meditation for high emotional eaters [45]), as well as more upstream approaches targeting the food environment at the population-level (e.g. restricting the number of takeaway outlets around schools [46]).

Our study has several strengths including having extensively measured diet, body weight, adiposity and the food environment. In particular, we used an objective imaging method to measure adiposity instead of BMI which is, among other things, unable to differentiate between fat mass and muscle mass [47]. Furthermore, we included a relatively large sample of individuals from multiple areas across Cambridgeshire, UK, and with characteristics that are broadly representative of the regional population. However, the sample may be less representative of other regions of the UK, particularly in terms of ethnic diversity. Given that our Fenland Study sample was constituted of relatively older adult participants, the current study findings may also not be generalizable to younger adults. Another limitation is the cross-sectional study design, which does not allow for strong inferences about causality. A third limitation includes the reliability and validity of some of the measures. While previous research found that the TFEQ-R18 has good reliability and validity [48], emotional eating scores may be subject to floor and ceiling effects as they are derived from only three items [14]. Furthermore, it is possible that we failed to capture some foods commonly consumed outside of the home (e.g. fried chicken and Asian dishes) resulting in an underestimation of takeaway consumption. It is also possible that we have misclassified foods such those bought from supermarkets, as takeaway foods, resulting in an overestimation of takeaway consumption. However, we found a positive association between our measure of takeaway consumption and the frequency of consuming take-away meals at home (data not shown). Moreover, similar results were found for both outcomes (takeaway consumption and body fat percentage), while any under- or overestimations of takeaway consumption are unlikely to have impacted the moderation of eating behaviours we observed. As discussed in previous work [7], another limitation is the temporal mismatch between data sources, arising from capture of food outlet data at only one time point (2011) within the period of participant data collection (2005–2015). Takeaway consumption and body fat percentage for some participants predated their takeaway outlet exposure, and it is unknown how long participants had been exposed to takeaway food outlets in their home environment. This is a common consideration in research of this type.

Besides longitudinal studies, further research is needed in different populations to confirm the current study results. This research may use other, more reliable or complementary, eating behaviour trait measurements such as the new version of the TFEQ (TFEQ-R21 [49]), which further improved TFEQ’s psychometric properties, or Ecological Momentary Assessments that are able to reduce biases associated with retrospective recall [50]. Future studies may also want to investigate the moderating role of other psychological factors such as external eating, emotional eating in response to positive emotions, emotional under-eating and food-responsiveness (e.g. as assessed by the Adult Eating Behaviour Questionnaire [51]). Studies may also include other outcomes such as general energy-dense food consumption.

## Conclusion

Individual differences in eating behaviour traits and exposure to takeaway outlets were both associated with takeaway consumption and adiposity. The evidence that individuals with certain eating behaviour traits are more susceptible to takeaway outlets was weak; there was no evidence that the association between takeaway outlet exposure and takeaway consumption or body fat varied by uncontrolled eating. While the magnitude of other effects were small, we found that the positive association between takeaway outlet exposure and body fat percentage was strongest for low emotional eaters, and the positive association between takeaway outlet exposure and takeaway consumption was strongest for those with high cognitive restraint scores. Our findings indicate that it is important to implement integrated strategies addressing both individual and environmental influences of dietary behaviours to tackle the current obesity epidemic.

## Supplementary information

Supplementary File

## Data Availability

While the Fenland Study (https://www.mrc-epid.cam.ac.uk/research/studies/fenland/) data is not publicly available for reasons of confidentiality, it may be available upon reasonable request by emailing datasharing@mrc-epid.cam.ac.uk.
