# Supplementary material for: The moderating role of eating behaviour traits in the association between exposure to hot food takeaway outlets and body fatness
Source: Int J Obes (Lond). Author manuscript; Available in PMC 2023 Jun 8. (PMC10212760; doi:10.1038/s41366-023-01290-9)
Supplement: Supplementary File [file EMS176250-supplement-Supplementary_File.docx]

**Supplementary file**

Supplementary Table 1. Characteristics of the Fenland cohort including the complete analytical sample as well as for the population with measured eating behaviour traits

|  | | **Complete Fenland sample (N=12,325)** | | **Analytical sample (N=4791)** | |
| --- | --- | --- | --- | --- | --- |
|  | | **N** | **Mean (SD), median; p25-p75, or N (%)** | **N** | **Mean (SD), median; p25-p75, or N (%)** |
| Age, years (mean (SD)) | | 12,325 | 48.6 (7.5) | 4791 | 51.0 (7.2) |
| Female (n (%)) | | 12,325 | 6636 (53.8%) | 4791 | 2581 (53.9%) |
| Age at highest educational qualification (SD) | | 12,329 | 18.9 (3.8) | 4767 | 19.0 (3.8) |
| Annual household income, £ (n (%)) | <20,000 | 12,085 | 1654 (13.8%) | 4704 | 629 (13.5%) |
|  | 20,000-39,999 |  | 4243 (35.4%) |  | 1512 (32.2%) |
|  | ≥40,000 |  | 6093 (50.8%) |  | 2544 (54.3%) |
| Occupational social class  (n (%)) | Working class | 11,663 | 2470 (21.2%) | 4778 | 980 (20.6%) |
|  | Intermediate |  | 2302 (19.7%) |  | 985 (20.7%) |
|  | Professional |  | 6891 (59.1%) |  | 2794 (58.7%) |
| Takeaway outlets in home neighbourhood, count (median (IQR)) | | 12,325 | 2; 1 - 14 | 4791 | 2; 0 – 12 |
| Supermarkets in home neighbourhood, count (median (IQR)) | | 12,325 | 1; 0 - 3 | 4791 | 1; 0 - 2 |
| Emotional eating, score ranging from 0-100  (mean SD)) | | 4791 | 34.7 (27.6) | 4791 | 34.7 (27.6) |
| Uncontrolled eating, score ranging from 0-100 (mean (SD)) | | 4791 | 29.9 (17.4) | 4791 | 29.9 (17.4) |
| Cognitive restraint, score ranging from 0-100 (mean (SD)) | | 4791 | 41.2 (19.6) | 4791 | 41.3 (19.6) |
| Takeaway food consumption, g/day (median (IQR)) | | 12,325 | 30.0; 17.5 - 47.5 | 4791 | 30.0; 17.5 – 47.5 |
| Body fat percentage (mean (SD)) | | 11,776 | 33.0 (9.2) | 4754 | 33.3 (9.2) |
| Body mass index (mean (SD)) | | 12,324 | 26.9 (4.8) | 4791 | 27.0 (4.8) |

Supplementary Table 2. Associations of exposure to takeaway outlets and eating behaviour traits with FMI and BMI in the imputed Fenland Study sample (n=4791)

|  | FMI | | BMI | |
| --- | --- | --- | --- | --- |
|  | B | 95% CI | B | 95% CI |
| Takeaway outlet exposure |  |  |  |  |
| Q1 (0) | Ref. | Ref. | Ref. | Ref. |
| Q2 (1-2) | 0.0 | -0.0; 0.0 | 0.2 | -0.2; 0.6 |
| Q3 (3-12) | **0.1** | **0.1; 0.1** | **1.2** | **0.8; 1.6** |
| Q4 (13-51) | **0.1** | **0.0; 0.1** | **1.0** | **0.3; 1.7** |
| Cognitive restraint | 0.0 | -0.0; 0.0 | **0.1** | **0.0; 0.2** |
| Emotional eating | **0.0** | **0.0;0.0** | **0.6** | **0.6; 0.7** |
| Uncontrolled eating | **0.1** | **0.1; 0.1** | **0.8** | **0.8; 0.9** |

Bold values are statistically significant at p<0.05

Eating behaviour trait scores range from 0-100 and are presented per 10 units

All models were adjusted for age, sex, household income, occupation, age at highest educational qualification and counts of supermarkets in home neighbourhoods

Abbreviations; FMI = Fat Mass Index, BMI = Body Mass Index, B = unstandardized beta regression coefficient, 95%CI = 95% confidence interval, Ref. = Reference

Supplementary Table 3. Associations of exposure to takeaway outlets and eating behaviour traits with consumption of takeaway food and body fat percentage in the unimputed Fenland Study sample

|  | Takeaway consumption^1^ | | Body fat %^2^ | |
| --- | --- | --- | --- | --- |
|  | B | 95% CI | B | 95% CI |
| Takeaway outlet exposure |  |  |  |  |
| Q1 (0) | Ref. | Ref. | Ref. | Ref. |
| Q2 (1-2) | 0.7 | -1.3; 2.7 | -0.2 | -0.4; 0.8 |
| Q3 (3-12) | **3.0** | **0.8; 5.2** | **1.8** | **1.2; 2.5** |
| Q4 (13-51) | **4.7** | **1.0; 8.5** | **2.0** | **0.9; 3.1** |
| Cognitive restraint | **-2.2** | **-2.6; -1.9** | **0.2** | **0.1; 0.3** |
| Emotional eating | **0.6** | **0.4; 0.9** | **0.9** | **0.8; 1.0** |
| Uncontrolled eating | **1.3** | **0.9; 1.7** | **1.1** | **1.0; 1.2** |

Bold values are statistically significant at p<0.05

Eating behaviour trait scores range from 0-100 and are presented per 10 units

All models were adjusted for age, sex, household income, occupation, age at highest educational qualification and counts of supermarkets in home neighbourhoods

Abbreviations; B = unstandardized beta regression coefficient, 95%CI = 95% confidence interval, Ref. = Reference

^1^ N=4609

^2^ N=4509

Supplementary Table 4. Mean difference in takeaway consumption or body fat percentage by takeaway outlet exposure and eating behaviour traits in the imputed Fenland study sample (n=4791)

| Takeaway outlet exposure | Eating behaviour traits | Mean difference in TAC | 95%CI |
| --- | --- | --- | --- |
| Q1 | Low cognitive restraint | Ref | Ref |
| Q2 | Low cognitive restraint | 1.6 | -1.1; 4.4 |
| Q3 | Low cognitive restraint | 2.2 | -0.8; 5.1 |
| Q4 | Low cognitive restraint | 2.1 | -2.1; 6.2 |
| Q1 | Mean cognitive restraint | Ref | Ref |
| Q2 | Mean cognitive restraint | 0.4 | -1.6; 2.3 |
| Q3 | Mean cognitive restraint | **2.5** | **0.4; 4.7** |
| Q4 | Mean cognitive restraint | **3.9** | **0.3; 7.5** |
| Q1 | High cognitive restraint | Ref | Ref |
| Q2 | High cognitive restraint | -0.9 | -3.7; 1.8 |
| Q3 | High cognitive restraint | 2.9 | -0.0; 5.9 |
| Q4 | High cognitive restraint | **5.7** | **1.5; 9.9** |
| Takeaway outlet exposure | Eating behaviour traits | Mean difference in BF% | 95%CI |
| Q1 | Low emotional eating | Ref | Ref |
| Q2 | Low emotional eating | 0.2 | -0.6; 1.0 |
| Q3 | Low emotional eating | **1.8** | **0.9; 2.6** |
| Q4 | Low emotional eating | **2.8** | **1.6; 4.0** |
| Q1 | Mean emotional eating | Ref | Ref |
| Q2 | Mean emotional eating | 0.3 | -0.2; 0.9 |
| Q3 | Mean emotional eating | **1.9** | **1.3; 2.5** |
| Q4 | Mean emotional eating | **2.2** | **1.1; 3.2** |
| Q1 | High emotional eating | Ref | Ref |
| Q2 | High emotional eating | 0.5 | -0.3; 1.3 |
| Q3 | High emotional eating | **2.0** | **1.2; 2.9** |
| Q4 | High emotional eating | **1.5** | **0.3; 2.7** |

Bold values are statistically significant at p<0.05

All models were adjusted for age, sex, household income, occupation, age at highest educational qualification and counts of supermarkets in home neighbourhoods

Abbreviations; 95%CI = 95% confidence interval, Ref. = Reference, BF% = Body fat percentage, TAC = Takeaway consumption
